# Meeting of the Rock Island County Medical Society

**Published:** 1857-09

**Authors:** W. F. Cady

**Affiliations:** Secretary


### PROCEEDINGS OF MEDICAL SOCIETIES.

*Meeting of the Rock Island County Medical Society.*

Pursuant to notice, the first quarterly meeting of the society was held at the office of Drs. Truesdale and Knox, Dr. Calvin Truesdale presiding.

A larger number of members were present than at any previous meeting; and the number was increased by a delegation from the Scott County Medical Society.

After the reading and approval of the minutes of last meeting, the committee appointed to confer with the Davenport Medical Society, with reference to union meetings, reported that arrangements had been made by which members of one society were to be received as honorary members of the other. On motion, the committee's report was adopted, when the following named gentlemen were admitted as members of the Rock Island Medical Society, without the privilege of ballot:

Drs. Thomas J. Saunders; J. W. H. Baker; Lewis F. Pelton, and W. M. Line, all of Scott County Medical Society.

Dr. O. P. S. Plummer, of Moline, was introduced, and admitted to membership.

Dr. P. Gregg, late president of the society was called out, and after expressing regrets that he was unable to be present at the annual meeting, proceeded to deliver his address prepared for that occasion. At the close of his reading, several members took occasion to make complimentary remarks for the enjoyment the address had afforded them, after which, as expressive of its approbation of the same, the society adopted the following:

*Resolved*, That our late president, Dr. P. Gregg, is entitled to our thanks for his highly profitable and interesting address; that a committee of three be appointed by the chair, to request a copy of the same for publication, to be preserved with the constitution and by-laws, as the annual address of the president of the first medical society organized in Rock Island County, Illinois.

Drs. Sam. K. Sharpe, W. A. Knox and J. R. Hayes were named as such committee.

Having been appointed to write a dissertation, for the present meeting, Dr. Sharpe read a learned and interesting article on the diagnosis, cause and treatment of *menorrhagia*, for which the society adopted a vote of thanks.

Dr. M. H. Carpster was appointed as essayist for the semiannual meeting, when Dr. Gregg moved that further business be deferred to a more convenient season, as the hour was near at hand, when he hoped to meet the professional brethren present, at his residence and dinner table; suggesting, moreover, that a carriage was in waiting to convey the company to the point proposed, and he trusted that members of the society would lose as little time as possible, but jump into the omnibus "and all take a ride." A proposition, at once so reasonable and disinterested, no one seemed inclined to oppose, and the society accordingly adjourned, to meet, in "*omnibus*."

W. F. CADY, Secretary.

